# Development and testing of the BONES physical activity survey for young children

**DOI:** 10.1186/1471-2474-11-195

**Published:** 2010-08-31

**Authors:** Christina D Economos, Erin Hennessy, Jennifer M Sacheck, M Kyla Shea, Elena N Naumova

**Affiliations:** 1 John Hancock Research Center on Physical Activity, Nutrition, and Obesity Prevention, Tufts University Friedman School of Nutrition Science and Policy, 150 Harrison Avenue, Boston, Massachusetts, USA; 2 Wake Forest University School of Medicine, Medical Center Boulevard, Winston-Salem, North Carolina, USA; 3 Tufts University School of Medicine, 136 Harrison Avenue Boston, Massachusetts, USA

## Abstract

**Background:**

Weight-bearing and high intensity physical activities are particularly beneficial for stimulating bone growth in children given that bone responds favorably to mechanical load. While it is important to assess the contribution and impact of weight-bearing physical activity on health outcomes, measurement tools that quantify and provide information on these activities separately from overall physical activity are limited. This study describes the development and evaluation of a pictorial physical activity survey (PAS) that measures children's participation and knowledge of high-intensity, weight-bearing ("bone smart") physical activity.

**Methods:**

To test reliability, two identical sets of the PAS were administered on the same day to 41 children (mean age 7.1 ± 0.8 years; 63% female) and compared. To test validity, accelerometry data from 40 children (mean age 7.7 ± 0.8 years; 50% female) were compared to data provided by the PAS. Agreements between categorical and ordinal items were assessed with Kappa statistics; agreements between continuous indices were assessed with Spearman's correlation tests.

**Results:**

The subjects produced reliable results in all 10 physical activity participation items (κ range: 0.36-0.73, all p < 0.05), but less reliable in answering if the physical activities were "bone smart" (κ range: -0.04-0.66). Physical activity indices, including metabolic equivalent time and weight-bearing factors, were significant in test-retest analyses (Spearman's *r *range: 0.57-0.74, all p < 0.001). Minutes of very vigorous activity from the accelerometer were associated with the self-reported weight-bearing activity, moderate-high, and high activity scores from the PAS (Spearman's *r *range: 0.47-0.48, all p < 0.01). However, accelerometer counts, counts per minute, and minutes of moderate-vigorous and vigorous activity were not associated with the PAS scores.

**Conclusions:**

Together, the results of these studies suggest that the PAS has acceptable test-retest reliability, but limited validity for early elementary school children. This survey demonstrates a first step towards developing a questionnaire that measures high intensity, weight-bearing activity in schoolchildren.

## Background

The 2008 Physical Activity Guidelines for Americans recommend that children participate in bone strengthening physical activity at least three days of the week [1]. This recommendation is based on numerous studies demonstrating that weight-bearing activities have a greater effect on bone mineral accretion than do weight-supported activities (e.g., bicycling, swimming) and may be more effective in reducing future risk of osteoporosis [2]. Even with these recommendations in place and the known benefits of exercise, American children do not get enough physical activity [3]. Intervention studies addressing these issues in young children remain a critical target of investigation.

To evaluate physical activity interventions aimed at preventing osteoporosis, it is critically important that researchers and practitioners have access to accurate, yet practical instruments. In general, there are a variety of methods to measure physical activity, which all offer advantages and disadvantages [4]. The choice of an instrument is dependent on the study purpose, design, resources, and participant characteristics [5]. Accelerometers (small computerized devices worn on the hip, ankle, or wrist) are a popular method of choice to provide an objective assessment of physical activity and have been used extensively with children. They are also being used in studies examining bone-related outcomes [6] given that accelerometers are able to measure weight-bearing physical activity [7]. However, the high cost and logistics of operation limit their widespread use in large community-based studies.

Many field trials utilize self-report questionnaires to assess physical activity [8]. In general, using these instruments with children in the 4^th ^grade or above has shown acceptable validity while other studies involving younger children have demonstrated mixed results. One possible explanation for the discrepancy in these findings is that most child self-report questionnaires (e.g. activity checklists) ask subjects to recall usual activity for periods longer than seven days, which generally results in lower validity coefficients. Studies with shorter recall periods have demonstrated more positive results [9]. As an alternative, some studies involving young children have attempted to use parental report of child physical activity, but their results suggest that this may be problematic [10]. Despite these measurement challenges with young children, there remains a need to develop simple physical activity assessment tools that can be used directly with children in larger, community-based research studies [11]. In addition, the ability to capture information from children on physical activity that enhances bone health is extremely limited.

The BONES (Beat Osteoporosis: Nourish and Exercise Skeletons) Project was a three-year randomized controlled trial in after school programs. BONES was designed to maximize bone development, bone quality, muscular strength, and calcium intake in 1^st^-3^rd ^grade children (n = 1400) attending after school programs in diverse communities across Massachusetts and Rhode Island. Children participated in weekly nutrition education lessons aimed at improving knowledge of "bone smart" foods (i.e. calcium-rich) and "bone smart" activities (i.e. weight-bearing). Children also engaged in daily physical activity lessons which ranged from short, 10-min jumping activities to longer, 30-min moderate-vigorous intensity activity sessions. At the study onset, there was no self-report questionnaire available for measuring weight-bearing physical activity in early elementary school children that did not require parental assistance or that quantified weight-bearing physical activity in children as young as six years. The purpose of this paper is to describe the development, test-retest reliability, and criterion validity of the BONES physical activity survey (PAS).

## Methods

### Development and Protocol of the BONES PAS

In an effort to adequately evaluate the role of weight-bearing physical activity in bone mineral accrual during the longitudinal study, the research team developed a questionnaire that measured both participation in and knowledge of weight-bearing physical activity. Knowing that measurement of physical activity in children under 10 years of age is challenging [8], the study team employed several techniques suggested by other researchers to develop the BONES PAS. For instance, some have suggested that the most promising physical activity self-report questionnaires for children restrict recall to just the previous day's activities due to children's limited ability to understand time orientation; however, few have utilized this technique [12]. Additionally, given that most questionnaires assess overall participation in physical activity, focusing on one type of activity (e.g. weight-bearing) may also aid children's recall ability. In the dietary assessment literature, others have recommended using creative methods that rely on cues and prompts to add context and aid recall in children, which may provide data of sufficient accuracy to be used to assess a specific aspect of children's behavior [13].

The goal of the BONES PAS was to evaluate high intensity, weight-bearing physical activities (i.e. running, jumping). It was based on extensive qualitative work investigating the activity habits of young children. Focus groups were held with 6-9 year old children, the literature was reviewed, and physical education specialists were consulted to identify common weight-bearing activities that children engage in on a regular basis. Research staff also directly observed children playing in after school settings on multiple occasions during the pilot phase of the BONES intervention. The need to quantify weight-bearing physical activity was balanced against the cognitive limitations of children (i.e. short attention span, inability to accurately estimate time). Given the age of the target population, the picture-sort technique was chosen as an appropriate method for this population.

**Figure 1:**
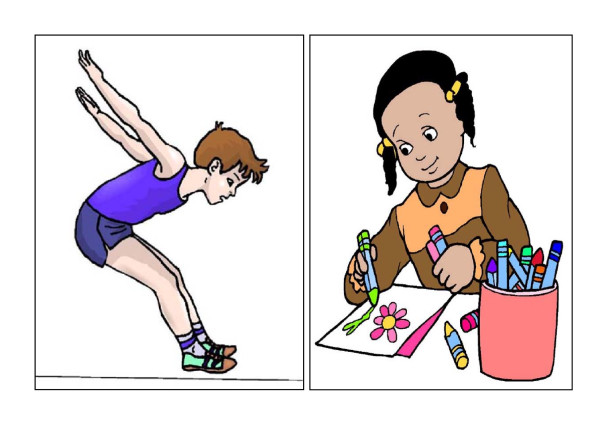
**Examples of picture cards used in the BONES Physical Activity Survey**. a) Jumping b) Drawing/Coloring.

The final version of the BONES PAS contained picture cards of children performing common activities: running, bike riding, jumping, hopping, skipping, playing on a jungle gym, swinging, watching television (TV), drawing or coloring, and playing video/computer games (see Figure 1 for two examples). The picture cards also represent common activities that children perform either as a single activity (i.e. jumping rope) or as part of other activities (i.e. jumping is incorporated into gymnastics). Pictures were created from cartoon-like images to enhance the age-appropriateness of the survey and to minimize any gender or cultural bias. Low-impact activities (i.e. drawing/coloring) were included in an effort to mask the true purpose of the instrument, yet were common for children to do.

To assess participation, the BONES PAS was administered in the following way. Children were first given the physical activity pictures, one at a time, and asked to describe what the person(s) in the picture was(were) doing. After it was clear to the interviewer that the child understood the activities represented by the pictures, the child was then given three color-coded placemats that had the words "yes", "no", and "I don't know". Children were instructed to sort each picture card into one of the three piles based on whether they did ("yes"), did not ("no"), or did not remember ("I don't know") doing the pictured activity over the previous two days ("yesterday" and "the day before yesterday"). For the knowledge component, the same picture cards were used again with three different color-coded placemats that had the words "good for building bones," "not good for building bones," and "I don't know". Each child was instructed to sort the activity cards based on what they believed.

### Subjects

The test-retest reliability and criterion validity studies were conducted simultaneously with recruited participants from the larger BONES study. Forty-one children (63% female), mean age 7.1 ± 0.8 years, participated in the reliability portion from the fall of 2000 through the spring of 2002. Forty children (50% female), mean age of 7.7 ± 0.8 years, participated in the validation study, which took place from the spring of 2001 through the fall of 2002. No child who was part of the reliability study was part of the validation study. Participation was voluntary. Parents gave their signed, informed consent and children over the age of seven years gave their written assent. All procedures were approved by the Institutional Review Board of Tufts University and met standard ethical procedures regarding the involvement of children in research.

### Physical activity by accelerometry

The Actigraph (Model 7164; Actigraph, LLC, Pensacola, FL) accelerometer has been previously investigated for its ability to detect high intensity, weight-bearing physical activity [7]. This monitor has been validated and used frequently in studies involving young children and is considered a good standard for validating self-report physical activity questionnaires [14,15].

Monitors were calibrated (using the manufacturer's calibrator, model CAL71) and initialized the day before they were distributed. Thirty-second epochs were specified. Trained staff met with participants at the after school program to instruct them on proper placement (over the right hip on an elasticized belt) and the importance of consistently wearing the monitor. Monitors were programmed to begin measuring at 6 a.m. the morning following distribution.

The children were instructed to wear the monitor during all waking hours with the exception of swimming and bathing. Parents were given and received instruction on how to maintain a detailed logbook to track their child's activity (including sedentary time) over the study period. The purpose of the logbooks was to complement the accelerometer data and provide the context and description for the type of activity children engaged in during the study period.

On the third day after distribution, research staff collected the monitors and logbooks from the participants at the after school program. Data from all monitors were subsequently downloaded (n = 40) and the two days corresponding to the PAS were analyzed using SAS (version 9.1; SAS Institute Inc., Cary, NC). Data reduction steps and inclusion/exclusion criteria were adapted from Masse [16] and the NHANES protocol [3]. For example, counts were first screened for spurious data including the affected values having an extended sequence of the maximum recordable value, counts beyond the biologically plausible range, or sequences of 60+ minutes in which activity never returned to zero. Second, a valid day was defined as having 80% of a standard day and a standard day was defined as the subject wearing the monitor for 70% of the time. Wear time was determined by subtracting non-wear time from 24 h. Non-wear was defined by an interval of at least 60 consecutive minutes of zero activity intensity counts, with allowance for 1-2 min of counts between 0 and 100.

The amount of physical activity as measured by accelerometer is presented as a sum of total counts across both data collection days and estimates of the time spent in physical activity according to count thresholds. Total counts evaluate the raw data provided by the accelerometer without the imposition of any external criteria other than determination of wear and nonwear time [3]. Time spent in physical activity of varying intensity levels (light, moderate, vigorous, very vigorous) is based on application of count thresholds corresponding to intensity-specific activity. Specific cut points relating the accelerometer counts/minute to METS were used according to Freedson [17] and adapted 30-s epoch (dividing the counts/minute cut-points by two). Data was also summed for any activity greater than moderate intensity for a total of moderate-vigorous physical activity (MVPA) and vigorous physical activity (VPA). Participants who did not wear the monitor for the two days prior to the PAS were excluded from all subsequent analysis (n = 6). Data for participants with valid days are described in the analyses below (n = 34; 50% female).

### Physical activity by self-report: BONES PAS

Since participants were recruited from after school programs, the BONES PAS was administered in this setting. For the reliability study, two trained research assistants administered the PAS independently, once to each child on the same day, at least 1-2 hours apart (referred to as T1 and T2). For the validity study, trained staff administered the PAS at the time the monitor was collected from the participant (the third day after distribution) so that the PAS reflected the activities that were captured by the accelerometer over the previous two days.

To score the BONES PAS, a weight-bearing factor (WBF) [18] and metabolic equivalence time (MET) [19,20] values were assigned to each activity (Table 1). A WBF score was calculated by adding the weight-bearing factor of the reported weight-bearing activities (for example, jumping WBF = 3). To calculate a moderate-high MET score MET values of all activities with a MET value ≥3 (moderate) were added to MET values of all activities with a MET value ≥6 (high). For physical activity knowledge, each correct response was scored as 1 and all incorrect scores including the "don't know" responses were scored as 0. The total number of correct responses was summed across all activities to provide the knowledge score.

**Table 1:** Assigned MET and WBF scores to each activity from the BONES physical activity survey (PAS)

Activity	MET	WBF
Jumping	10	3
Running	8	1
Biking	7	0.5
Hopping	5	1
Playing on Playground	5	1
Skipping	5	1
Computer/Video games	2	0
Swinging	2	0
Drawing	1.8	0
Watching TV	1	0

*Note*. MET = Metabolic equivalent time; MET values [19,20]; WBF = weight-bearing factor; WBF scores [18].

### Statistical analysis

To test the reliability of the categorical items in the PAS, such as the answer sets consisting "yes", "no", and "I don't know", Cohen's Kappa (κ) statistic was used. As a guide for interpreting the Cohen's κ we used the ratings developed by Landis and Koch [21]: fair (κ = 0.21-0.40), moderate (0.41-0.60), substantial (0.61 to 0.80), and almost perfect (> 0.80). Spearman's correlations were used to assess test-retest reliability for T1 and T2 for each of the continuous indices, including weight-bearing score, moderate-high MET score, high MET score, and knowledge score.

For the validation study, Spearman's correlations were used to estimate the association between the self-reported activities and the objectively measured variables. We used a similar comparison study conducted by deRidder to reflect low, moderate, or high validity [18]. We also examined intensity-specific activity, but given the discrepancies between various cut-points in the literature we also examined absolute counts from the accelerometer. The response rate for the logbooks was poor (17 logbooks were not usable: 9 not returned, 2 returned blank, 3 missing data from one of the measurement days, and 3 with unidentifiable information) and were not used in the analysis. All analyses were performed for the group using SPSS (version 17.0; SPSS Inc., Chicago, IL). An alpha level of .05 was used for all statistical tests.

## Results

### Reliability study

For the questions in the physical activity participation component, percent agreement between T1 and T2 ranged from 61.0% (hopping) to 95.1% (watching TV) as shown in Table 2. Agreements in five activities (jumping, hopping, skipping, playing on playground, swinging) fell below 75% while the κ-statistic for these items indicated moderate reliability. The one exception to this was for hopping, which demonstrated only fair reliability. For the questions in the knowledge component, percent agreement ranged from 60.5% (hopping) to 97.4% (running). The κ-statistic ranged from fair (jumping, biking, hopping, skipping, video games, and watching TV) to substantial (running, drawing/coloring). Despite a high percent agreement (87.2%) for jumping, the κ-statistic was not significant. Table 3 shows the overall group characteristics and estimates of the Spearman's correlations for the physical activity participation and knowledge scores indicating that the BONES PAS demonstrated acceptable reliability for the survey outcomes (p < 0.001).

**Table 2:** Reliability for physical activity participation and knowledge of bone smart physical activities

	**Participation in physical activities (n = 41)**				**Knowledge in "bone smart" physical activities (n = 38)**			
		
**Activity**	**Frequency ^a^**		**% agreed**	**κ (95%CI)**	**Frequency ^b^**		**% agreed**	**κ (95%CI)**
						
	**Test**	**Re-test**			**Test**	**Re-test**		
	
**Jumping**	21/18/2	20/18/3	70.7	0.47 (0.24, 0.71)***	34/1/3	35/0/3	86.8	0.22 (-0.24, 0.69)
**Running**	29/12/0	33/7/1	75.6	0.36 (0.04, 0.67)*	36/0/2	37/0/1	97.4	0.66 (0.01, 1.3)***
**Bike riding**	21/20/0	19/19/3	85.4	0.73 (0.53, 0.92)***	2/34/2	0/37/1	89.5	0.17 (-0.08, 0.43)
**Hopping**	11/24/6	12/25/4	61.0	0.29 (0.03, 0.55)*	23/5/10	26/2/10	60.5	0.23 (-0.02, 0.47)
**Playing on Playground**	21/17/3	18/20/3	68.3	0.44 (0.18, 0.7)**	18/8/12	16/9/13	73.7	0.59 (0.37, 0.81)***
**Skipping**	9/29/3	13/26/2	73.2	0.44 (0.17, 0.71)**	26/4/8	24/4/10	68.4	0.37 (0.09, 0.65)**
**Computer/Video games**	14/22/5	16/21/4	75.6	0.58 (0.36, 0.79)***	31/2/5	31/2/5	68.4	0.00 (-0.29, 0.28)
**Swinging**	19/21/1	14/23/4	68.3	0.43 (0.19, 0.67)**	7/20/11	7/18/13	71.1	0.53 (0.3, 0.77)***
**Drawing/coloring**	28/8/5	31/7/3	80.5	0.56 (0.29, 0.82)***	19/8/10^d^	15/11/11^d^	75.7	0.62 (0.4, 0.85)***
**Watching TV**	39/2/0	37/3/1	95.1	0.65 (0.19, 1.11)***	37/0/1	33/0/5	89.5	0.30 (-0.17, 0.78)**

CI: Confidence interval; *: p < 0.05; **: p < 0.01; ***: p < 0.001

^a ^The numbers indicate the frequencies of yes/no/don't know

^b ^The numbers indicate the frequencies of correct/incorrect/don't know

^c ^The Kappa statistics (κ) was originally unavailable due to non-square contingency tables. Cases with a sample weight of 0.1 were added to recover the estimate

^d ^N = 37 due to one missing answer

**Table 3:** Spearman's correlation of reliability for physical activity participation and knowledge of bone smart physical activities

**Indicators**	**N**	**Mean ± Standard deviation**		**Median (lower & upper quartiles)**		***r_s _*(95% CI)^a^**
						
		**Test**	**Re-test**	**Test**	**Re-test**	
	
MET Score (moderate-high)	41	19.37 ± 10.59	19.80 ± 11.42	20.0 (13.0, 29.0)	20.0 (13.0, 30.0)	0.74 (0.56, 0.85)***
MET Score (high)	41	14.37 ± 7.73	14.56 ± 7.90	15.0 (8.0, 18.0)	15.0 (8.0, 21.5)	0.57 (0.32, 0.75)***
WBF Score	41	3.50 ± 2.19	3.55 ± 2.38	4.0 (1.5, 5.5)	2.5 (1.5, 5.8)	0.71 (0.51, 0.83)***
Total correct answers in PA knowledge	38	5.05 ± 1.58	5.03 ± 1.46	5.0 (4.0, 6.0)	5.0 (4.0, 6.0)	0.73 (0.53, 0.85)***

MET: Metabolic equivalent time; WBF: Weight-bearing factor; *r_s_*: Spearman's correlation coefficient

***: p < 0.001

^a ^CI: Confidence Interval; derived using Fisher's *z_r _*transformation, two-tailed

### Validation study

Table 4 summarizes the mean weight-bearing, moderate-high and high MET scores from the PAS and the accelerometry data expressed as a sum of both days represented by the PAS. On average, children engaged in over two hours per day above the moderate threshold for activity. The mean daily accelerometer wear time was 12.5 hours and mean counts per minute was 854.69 over the two day monitoring period. Each of the calculated PAS scores (weight-bearing, moderate-high and high MET) was associated with the amount of very vigorous activity captured by the accelerometer (p < 0.01; Table 5). No statistically significant relationship was found between the PAS and the total counts, moderate-vigorous or vigorous activity from the accelerometer.

**Table 4:** Descriptive statistics of the BONES physical activity survey and accelerometry data (n = 34)

*Indicators*	Mean ± SD
**BONES physical activity survey (PAS):**	
Moderate-high MET score	22.62 ± 10.07
High MET score	16.15 ± 7.41
Weight-bearing score^a^	4.28 ± 2.19
**Accelerometry (Sum of two days):**	
Total counts	633302.50 ± 179639.90
Total minutes worn	1494.03 ± 171.94
Counts/minute	854.69 ± 232.90
**Minutes of:**	
**I: **Moderate activity (3-6 METs)	260.71 ± 77.18
**II: **Vigorous activity (6-9 METs)	20.25 ± 12.96
**III: **Very vigorous activity (> 9 METs)	5.49 ± 6.36
**I + II + III: **Total moderate-vigorous PA; (MPVA; ≥ 3 METs)	286.44 ± 88.09
**II + III: **Total vigorous PA (VPA; ≥ 6 METs)	25.74 ± 17.54

PAS: MET: Metabolic equivalent time; SD: Standard deviation; PA: Physical activities

^a ^Weight-bearing score was calculated by adding the weight-bearing factor (WBF) of the reported activities (See Table 1)

**Table 5:** Spearman's correlations between the BONES PAS and the Actigraph 7164 accelerometer

	***r_s _*(95% CI)^a^**		
	
**Accelerometer data**	**BONES PAS Scores**		
			
	**Moderate-high MET** **(3-6 METs)**	**High MET** **(6-9 METs)**	**Weight-bearing Factor**
		
Counts, total	0.24 (-0.08, 0.51)	0.2 (-0.12, 0.48)	0.23 (-0.09, 0.51)
Counts, per minute	0.27 (-0.05, 0.54)	0.25 (-0.07, 0.52)	0.26 (-0.06, 0.53)
Vigorous	0.21 (-0.11, 0.49)	0.23 (-0.09, 0.51)	0.21 (-0.11, 0.49)
Very vigorous (≥ 9 METs)	0.47 (0.19, 0.68)**	0.48 (0.2, 0.69)**	0.48 (0.2, 0.69)**
Total vigorous physical activity (≥ 6 METs)	0.25 (-0.07, 0.52)	0.27 (-0.05, 0.54)	0.24 (-0.08, 0.51)
Total moderate-vigorous physical activity (≥ 3 METs	0.17 (-0.15, 0.46)	0.13 (-0.19, 0.42)	0.16 (-0.16, 0.45)

MET: Metabolic equivalent time; *r_s_*: Spearman's correlation coefficient

**: p < 0.01

^a ^CI: Confidence Interval; derived using Fisher's *z_r _*transformation, two-taile

## Discussion

The BONES PAS is a unique self-report survey that was developed to focus on high intensity and weight-bearing activity in early elementary school-aged children without parental assistance. No other published physical activity surveys for children have focused on this type of activity. The survey demonstrated acceptable test-retest reliability for both participation in and knowledge of weight-bearing physical activity, but several specific items warrant further investigation. For instance, the percent agreement was highest for activities that are likely reflective of frequent and routine habits of children in this age group (i.e. watching TV, bike riding). Two items--hopping and skipping--were the two activities that were not as common for children to report participating in over the two day recall period. However, only hopping demonstrated a low percent agreement and fair reliability. This may be due to the uncertainty about what is meant specifically by hopping or due to the fact that bouts of hopping tend to be short, occur as part of other sporting activities, and may therefore be more difficult to recall. Several of the items demonstrated only fair reliability with respect to knowledge of weight-bearing physical activity. Overall, despite the similarity to jumping, fewer children were able to classify hopping as "bone smart". This may be reflective of children knowing that physical activity is good for the body but are unaware of the specific health benefits (i.e. good for bones), which may be driving them to answer the question differently at each time point. However, when these items are combined into the BONES PAS scores, the two time points demonstrated a moderately high association.

The PAS may provide valid, yet limited information for evaluating weight-bearing and very high intensity activity. It is important to assess very vigorous activity in children as high intensity that strains the musculoskeletal system is more important than the volume of activity for bone development [22]. However, the amount of very vigorous activity that children participate in represents a very small fraction of their overall activity, which may be difficult to detect with a survey and limits the usefulness of this instrument. The associations found between the BONES PAS and accelerometry are approximate to, but slightly lower than, the BONESTAAK study, which looked at weight-bearing physical activity with a slightly older (mean age 11 years) population [18]. In this study, de Ridder and colleagues [18] created a questionnaire to assess weight-bearing physical activity in children (8-14y) given that one did not exist to evaluate their intervention. Their tool, the Weight-Bearing Activity Questionnaire for Kids (WBAQK), was validated against the Caltrac™ accelerometer and demonstrated a higher association in their sample (r > 0.50) than what was found in this study. Other studies that have validated questionnaires estimating total activity against accelerometers have reported lower correlations (r = 0.27, p = 0.03 [12] and r = 0.34, p = 0.004 [23]). This evidence provides contextual support that the BONES PAS demonstrates moderate validity.

Despite a concerted effort to improve upon existing self-report questionnaires to optimize children's recall ability [11], the findings presented here may be indicative of difficulties collecting self-report data from young children. Children often struggle when using recall instruments: they tend to elide (i.e. merge together) experiences, have trouble remembering a whole day, have a poor sense of duration, have difficulty determining intensity of activity and often lack motivation to complete the task [22]. The BONES PAS differs from other surveys by restricting the recall period to two days in an attempt to capture habitual activity, focusing on type rather than amount of activity, and using pictures for children to report their activity habits. As such, other aspects of the study design may be driving our null results. For instance, the time resolution of the accelerometer is important when assessing activity relevant to bone density as this allows for short periods of intense activity to be captured [11].

Utilizing a 5 s or even 1 s epoch rather than 30 s may have provided us with a better picture of the child's true activity level [24] by more accurately capturing high intensity physical activity [25]. Unfortunately, at the time of the study, standard epoch lengths for measurement of activity in children typically did not span shorter than 1-minute intervals. At the time, the 30-second epoch length was still relatively new. Future physical activity survey validation work should consider the shorter one to five second epoch lengths.

Additionally, the selection of an appropriate criterion measure against which to validate our survey was difficult due to the absence of a universally accepted gold standard for measuring physical activity participation [26]. Since the PAS was designed to capture habitual participation in weight-bearing physical activity, direct observation would not have been possible since our observation period would have been restricted to the after school setting during weekdays only. Although the choice of accelerometers did limit our analytic ability, it is one of the few objective measures that have been used in other studies measuring weight-bearing activity [7]. Tracking activity through logbooks to determine mode of activity may have provided additional insight; however, we were unable to collect reliable information using this procedure. Currently there are new methodologies being developed with accelerometers that may allow determination of activity type from examining the count data [27] in addition to intensity and duration. Although this work is in its infancy, it may soon help to resolve issues that hamper these types of studies. Our analysis was also hindered at the time by a lack of energy cost data for children performing adult activities or child-specific activities [11]. The sample size was also limited and slightly lower than other similar studies [9,23]. Most of the Spearman's correlation coefficients in Table 5 are in the range of 0.20 and 0.25. In order to detect a significant Pearson's correlation of 0.25 with alpha set at 0.05 and power at 0.8, a sample size of 98 is needed. For non-parametric technique such as Spearman's correlation, the number could have been lower, but undoubtedly a larger sample may have allowed us to better assess which aspects were driving the null results.

Despite extensive pilot work to develop the BONES PAS, it may not adequately allow children to recall their physical activity participation. Children's activity tends to be more sporadic and spontaneous in nature. As such they may not recall doing certain activities that are incorporated into more structured events (i.e. sports). For instance, children may run or jump as an independent activity or incorporate this behavior into other activities such as games and sports. However, it is unclear if children are accurately recalling an activity such as jumping if this activity only occurred intermittently during a soccer game; yet, the impact of this would have been assessed by the accelerometer. In addition, time constraints of conducting the study within an after school setting limited our study design to test the children no more than 2 hours apart. We recognize that this is a limitation given that children may be able to recall their answers from T1. If the time between administrations of the tests had been extended then the subject could change their relative amount of activities performed, which may have complicated questioning. The time allocation used in this study has also been employed successfully by other investigators [28].

Future testing of the BONES PAS and other efforts to measure bone-building physical activity should include cognitive interviewing of children to determine how they recall these activities. Additionally, other changes could be incorporated into study methodology that could potentially help to improve a child's recall ability. For instance, orienting children to specific time periods of the day that are relevant to them (i.e. before, during, or after school) may help them to remember what activities they performed rather than simply asking if the child performed the activity at all during the day. Despite extensive qualitative work, there may be other physical activity options that the PAS is missing that could also have an impact on bone (i.e. dancing). Small changes to the BONES PAS may improve the utility of the tool significantly.

## Conclusions

It is well established that increased participation in weight-loading physical activity in childhood positively influences bone health [29,30]. Interventions that target this health behavior early in life are important for the prevention of osteoporosis, and it is also critically important that researchers and practitioners have access to accurate, yet practical instruments to evaluate these interventions. To our knowledge, no self-report questionnaire is available for measuring weight-bearing physical activity in large field trials targeting early elementary school children. The results of this study suggest that the BONES PAS has acceptable test-retest reliability, but limited validity for early elementary school children. This survey has potential for measuring high intensity, weight-bearing activity but further investigation is warranted. The BONES PAS provides an opportunity for further research that could eventually result in a reliable, valid, and versatile self-report measure of weight-bearing physical activity that is specifically designed for children.

## Abbreviations

BONES: Beat Osteoporosis: Nourish and Exercise Skeletons; MET: metabolic equivalent time; MVPA: moderate-to-vigorous physical activity; PAS: physical activity survey; VPA: vigorous physical activity; WBF: weight-bearing factor

## Competing interests

The authors declare that they have no competing interests.

## Authors' contributions

CDE, KS, EN participated in the conception and design of the study. EH, KS, and EN performed the statistical analysis. KS carried out the design and coordination of the reliability and validation studies while CDE, EH, and KS participated in the data collection. CDE, KS, EH, and JMS drafted the manuscript. All authors read and approved the final manuscript.

## Pre-publication history

The pre-publication history for this paper can be accessed here:

http://www.biomedcentral.com/1471-2474/11/195/prepub
